# Molecular characterization of Mycobacterium abscessus strains isolated from a hospital outbreak.

**DOI:** 10.3201/eid0605.000524

**Published:** 2000

**Authors:** L A Raman, N Siddiqi, M Shamim, M Deb, G Mehta, S E Hasnain


Three of the four HPS samples previously confirmed by the ALI in São Paulo tested positive by our ELISA. Of the remaining 12 suspected HPS cases assayed, three were positive. Two of these three were later confirmed as positive by the ALI (Luiza Teresinha Madia de Souza, ALI, pers. comm.) Thus, we report three previously unconfirmed HPS cases, one fatal, in the Ribeirão Preto area between May 1998 and August 1999.

Since the rodent reservoir is not known and the virus has not been isolated, rodent capture is currently being conducted in areas where human infections have been found. In addition, positive cases are being retrospectively investigated.

Thanks to Thomas Ksiazek for providing recombinant SNV antigen and positive serum and to Robert Shope for his assistance.

The study was partially funded by grants from the National Institutes of Health, Bethesda, Maryland, USA, and Fundação de Amparo à Pesquisa do Estado de São Paulo, Brazil.

**Ryan Holmes,* Rodrigo Boccanera,† Luiz Tadeu M. Figueiredo,† Silvia Regina Mançano,‡ Clodoaldo Pane§**
*University of Texas Medical Branch at Galveston, University of Texas School of Public Health at Houston, Texas, USA; †Unidade Multidepartamental de Pesquisa em Virologia da Faculdade de Medicina de Ribeirão Preto-USP, Brazil; ‡Centro de Saúde de Guariba, São Paulo State, Brazil; and §Centro de Saúde de Jardinópolis, São Paulo State, Brazil.

### References

1. Figueiredo LTM, Moreli ML, Almeida VSO, Félix PR, Bruno, JC, Ferreira IB, et al. Hantavirus pulmonary syndrome (HPS) in Guariba, SP, Brazil. A report of 2 cases. Rev Inst Med Trop Sao Paulo 1999; 41:131-7.
2. Johnson AM, de Souza LTM, Ferreira IB, Pereira LE, Ksiazek TG, Rollin PE, et al. Genetic investigation of novel hantaviruses causing fatal HPS in Brazil. J Med Virol 1999;59:527-35.
3. Ferreira MS, Nishioka SA, Santos TL, Santos RP, Santos PS, Rocha A. Hantavirus pulmonary syndrome in Brazil: clinical aspects of three new cases. Rev Inst Med Trop Sao Paulo 2000;42:41-6.
4. Da Silva MV, Vasconcelos MJ, Hidalgo NT, Veiga AP, Canzian M, Marotto PC, et al. Hantavirus pulmonary syndrome: report of the first three cases in São Paulo, Brazil. Rev Inst Med Trop Sao Paulo 1997;39:231-4.
5. Peters CJ. Emerging infections: hantavirus pulmonary syndrome in the Americas. Washington: ASM Press;1998.
6. Zeitz PS, Butler JC, Cheek JE, Samuel MC, Childs JE, Shands LA, et al. A case-control study of hantavirus pulmonary syndrome during an outbreak in the southwestern United States. J Infect Dis 1995;171:864-70.

### Molecular Characterization of *Mycobacterium abscessus* Strains Isolated from a Hospital Outbreak

**To the Editor:** In recent years there have been several reports of sporadic and epidemic hospital-acquired infections caused by rapidly growing mycobacteria, namely, *Mycobacterium abscessus*, *M. chelonae*, and *M. fortuitum*. *M. abscessus* has been well documented as a cause of cutaneous and soft tissue infections and has been implicated in chronic ear infections, bacteremia associated with hemodialysis equipment, and peritoneal dialysis–related infections (1).

Differentiating mycobacteria to the species level is difficult because of the diversity of available techniques and the time required for full identification. A rapid method based on the evaluation of the gene coding for the 65-kDa heat shock protein, which contains epitopes both unique and common to various species of mycobacteria, has been reported (2). A 383-bp sequence situated at the amino terminus of this 65-kDa antigen (3) has been shown to be conserved among several species of mycobacteria. Reports based on polymerase chain reaction (PCR) amplification and DNA sequencing (4) show species-specific polymorphism at the nucleotide level within this region (5). The conserved nature of this gene allows differentiation of mycobacteria within 1 day by restriction enzyme digestion of PCR products obtained by using primers common to all mycobacteria.

From August through December 1995, postoperative wound infections developed in 45 patients in the pediatric surgery unit of Kalawati Saran Children's Hospital, New Delhi, India; 42 were day-care patients, and 3 were inpatients who had undergone major surgery. Thirty-two clinical samples (pus and exudate) were tested for acid-fast bacilli by the Ziehl-Neelson method; the same smear samples were cultured on Lowenstein-Jensen slants and examined for growth daily for 4 days and thereafter twice a week for 8 weeks. The organism was identified biochemically (i.e., by the niacin and nitrate production test) as *M. abscessus*.

The genomic DNA from the culture of mycobacterial isolates was extracted by standardized protocol (6) and subjected to PCR-restriction enzyme pattern analysis (PRA) (2). Primers TB11 (5'-ACC AAC GAT GGG GTG TGT CCA T) and TB12 (5'-CTT GTC GAA CCG CAT ACC CT) amplified a 439-bp fragment between positions 398 and 836 of the published gene sequence for 65-kDa heat shock protein (3). The PCR products were then digested separately by using restriction enzymes *Bst* EII and *Hae*III. The digests were fractionated on nondenaturing 10% polyacrylamide gel. The *Bst* EII pattern generated during PRA yielded two 235/210-bp bands similar to the patterns attributed to *M. chelonae* subsp. *abscessus* (2). The patterns displayed on *Hae*III digestion had distinctive 150/60-bp bands that were once again similar to the pattern attributed to *M. chelonae* subsp. *abscessus* (2). PRA results confirmed that the isolates were *M. abscessus*. The source of the outbreak was traced to the tap water in the operating room and to a defective autoclaving process (the result of a leaking vacuum pump and faulty pressure gauge in the autoclave).

This report highlights the role of rapidly growing mycobacteria in a water-related nosocomial outbreak. The PCR-PRA method promises to be a very rapid, economical, and universal system of identifying mycobacteria to the species level. This technique does not require hybridization to a panel of species-specific probes, which is a limitation of other PCR-based and hybridization methods for differentiating mycobacterial species. This method has the potential to be a useful diagnostic as well as epidemiologic marker for typing isolates of most mycobacteria during institutional outbreaks.

**Lakshmy Anantha Raman,* Noman Siddiqi, †‡ Mohammed Shamim,† Monorama Deb,* Geeta Mehta,* and Seyed Ehtesham Hasnain†‡**
*Lady Hardinge Medical College, New Delhi, India;
†National Institute of Immunology, New Delhi, India;
‡Centre for DNA Fingerprinting and Diagnostics, Hyderabad, India

### References

1. Hakim A, Hisam N, Reuman PD. Environmental mycobacterial peritonitis complicating peritoneal dialysis: three cases and review. Clin Infect Dis 1993;16:426-31.
2. Telenti A, Marchesi F, Balz M, Baally F, Bottger EC, Bodmer T. Rapid identification of Mycobacteria to the species level by polymerase chain reaction and restriction enzyme analysis. J Clin Microbiol 1993;31:175-8.
3. Shinnick T. The 65-kilodalton antigen of *Mycobacterium tuberculosis*. J Bacteriol 1987;169:1080-8.
4. Brisson-Noel A, Aznar C, Chureau C, Nguyen S, Pierre C, Bartoli M, et al. Diagnosis of tuberculosis by DNA amplification in clinical practice evaluation. Lancet 1991;338:364-6.
5. Hance A, Grandchamp B, Levy-Frebaault V, Lecossier D, Rauzier JJ, Bocart D, et al. Detection and identification of mycobacteria by amplification of mycobacterial DNA. Mol Microbiol 1989;3:843-9.
6. Siddiqi N, Shamim M, Jain NK, Rattan A, Amin A, Katoch VM, et al. Molecular anaylsis of multi-drug resistance in Indian isolates of *Mycobacterium tuberculosis*. Mem Inst Oswaldo Cruz 1998;3:589-94.

**Erratum Vol. 6, No. 4**

In the letter "First Report of Human Granulocytic Ehrlichiosis from Southern Europe (Spain)," by José Oteo et al., there are two citation errors on p. 431, column 2. The correct citations follow.

First paragraph, second sentence: "We used a set of primers based on the published sequence of the 16s rRNA of *E. phagocytophila* (E1: 5'-GGC ATG TAG GCG GTT CGC TAA GTT-3' and E2: 5'-CCC CAC ATT CAG CAC TCA TCG TTT A-3' (10)."

Second paragraph, second sentence: "The prevalence of *E. phagocytophila* genogroup in the tick *Ixodes ricinus* is high (24.1% of nymphs, determined by PCR) in La Rioja, and evidence of HGE infection in patients at risk has been reported (11)."

We regret any confusion this error may have caused.

